# Supplementary material for: scPADGRN: A preconditioned ADMM approach for reconstructing dynamic gene regulatory network using single-cell RNA sequencing data
Source: PLoS Comput Biol. 2020 Jul 27;16(7):e1007471. doi: 10.1371/journal.pcbi.1007471 (PMC7410337; doi:10.1371/journal.pcbi.1007471)
Supplement: S1 Text — By summing up all (genes and all) time points simultaneously, model can be transformed from vector form to matrix form. (PDF) [file pcbi.1007471.s001.pdf]

S1 Text: Derivation for Eq (6)

By summing up all m genes and all N time points simultaneously, we could transite vector form model to matrix form.

The optimization is

$$\min_{A(1),\cdots,A(N-1)} L = \sum_{\mathrm{t}=1}^{N-1}\sum_{i=1}^{m}\frac{1}{2}||(\Delta Y_i^T(\mathrm{t}))^{(exp)} - (\Delta Y_i^T(\mathrm{t}))^{(sim)}||_2^2.$$

With Eq (3) in main paper, we also have the following approximation:

$$\Delta Y_i^T(\mathrm{t}) = Y_i^T(\mathrm{t}+1) - Y_i^T(\mathrm{t}) \approx Y^T(\mathrm{t}) \cdot A_i^T(\mathrm{t}).$$

Then, the objective function L in optimization can be written as

$$L = \sum_{\mathrm{t}=1}^{N-1}\sum_{i=1}^{m}\frac{1}{2}||(\Delta Y_i^T(\mathrm{t}))^{(exp)} - (\Delta Y_i^T(\mathrm{t}))^{(sim)}||_2^2$$

$$= \frac{1}{2}\sum_{\mathrm{t}=1}^{N-1}\sum_{i=1}^{m}||[Y_i^T(\mathrm{t}+1) - Y_i^T(\mathrm{t}) - Y^T(\mathrm{t})A_i^T(\mathrm{t})||_2^2$$

$$= \frac{1}{2}\sum_{\mathrm{t}=1}^{N-1}||[(Y_1^T(\mathrm{t}+1) - Y_1^T(\mathrm{t})),\cdots,(Y_m^T(\mathrm{t}+1) - Y_m^T(\mathrm{t}))] - Y^T(\mathrm{t})[A_1^T(\mathrm{t}),\cdots,A_m^T(\mathrm{t})]||_F^2$$

$$= \frac{1}{2}\sum_{\mathrm{t}=1}^{N-1}||Y^T(\mathrm{t}+1) - Y^T(\mathrm{t}) - Y^T(\mathrm{t})A^T(\mathrm{t})||_F^2$$

$$= \frac{1}{2}\sum_{t=1}^{N-1}||Y(t+1) - Y(t) - A(t)Y(t)||_F^2.$$
