# Supplementary material for: scPADGRN: A preconditioned ADMM approach for reconstructing dynamic gene regulatory network using single-cell RNA sequencing data
Source: PLoS Comput Biol. 2020 Jul 27;16(7):e1007471. doi: 10.1371/journal.pcbi.1007471 (PMC7410337; doi:10.1371/journal.pcbi.1007471)
Supplement: S2 Text — PADMM is a variation of the ADMM. This text shows the process of preconditions. (PDF) [file pcbi.1007471.s002.pdf]

S2 Text: Details about PADMM

The PADMM is a variation of the alternating direction method of multipliers. Before introducing the PADMM, we present the ADMM algorithm for solving these three subproblems. The scaled ADMM is employed here since it is a more convenient form [1].

For subproblem 1, first, we convert it into ADMM form:

$$\min_{A(1),B(1),D(1)} \frac{1}{2}\|A(1)Y(1) - [Y(2) - Y(1)]\|_F^2 + \alpha\|B(1)\|_1 + \beta\|A(2)^k - D(1)\|_1$$

$$s.t.\ B(1) - A(1) = 0, D(1) - A(1) = 0.$$

Its augmented Lagrangian is

$$\begin{aligned} &L_\rho(A(1), B(1), D(1), U(1), W(1)) \\ &\quad = \frac{1}{2}\|A(1)Y(1) - [Y(2) - Y(1)]\|_F^2 + \alpha\|B(1)\|_1 + \beta\|A(2)^k - D(1)\|_1 \\ &\quad + \frac{\rho}{2}\|B(1) - A(1) + U(1)\|_F^2 - \frac{\rho}{2}\|U(1)\|_F^2 + \frac{\rho}{2}\|D(1) - A(1) + W(1)\|_F^2 \\ &\quad - \frac{\rho}{2}\|W(1)\|_F^2. \end{aligned}$$

The iterations are as follows:

$$\begin{cases} A(1)^{k+1} = [(Y(2) - Y(1)) \cdot Y(1)^T + \rho^k(B(1)^k + U(1)^k + D(1)^k + W(1)^k)] \cdot [Y(1)Y(1)^T + 2\rho^k I]^{-1} \\ B(1)^{k+1} = S_{\alpha/\rho^k}(A(1)^k - U(1)^k) \\ U(1)^{k+1} = U(1)^k + B(1)^k - A(1)^k \\ D(1)^{k+1} = S_{\beta/\rho^k}(A(1)^k - W(1)^k - A(2)^k) + A(2)^k \\ W(1)^{k+1} = W(1)^k + D(1)^k - A(1)^k, \end{cases}$$

where the soft thresholding operator S is defined as

$$S_\kappa(a) = \begin{cases} a - \kappa & a > \kappa \\ 0 & |a| \le \kappa. \\ a + \kappa & a < -\kappa \end{cases}$$

For subproblem 2, we convert it into ADMM form:

$$\min_{A(t),B(t),C(t),D(t)} \frac{1}{2}\|A(t)Y(t) - [Y(t+1) - Y(t)]\|_F^2 + \alpha\|B(t)\|_1$$

$$+\beta\|A(t+1) - C(t)\|_1 + \beta\|D(t) - A(t-1)\|_1$$

$$s.t.\ B(t) - A(t) = 0, C(t) - A(t) = 0, D(t) - A(t) = 0.$$

Its augmented Lagrangian is

$$
\begin{aligned}
&L_\rho(A(t),B(t),C(t),D(t),U(t),V(t),W(t)) \\
&\qquad = \frac{1}{2}\|A(t)Y(t)-[Y(t+1)-Y(t)]\|_F^2 + \alpha\|A(t)\|_1 + \beta\|A(t+1) \\
&\qquad - A(t)\|_1 + \beta\|A(t)-A(t-1)\|_1 + \frac{\rho}{2}\|B(t)-A(t)+U(t)\|_F^2 \\
&\qquad - \frac{\rho}{2}\|U(t)\|_F^2 + \frac{\rho}{2}\|C(t)-A(t)+V(t)\|_F^2 - \frac{\rho}{2}\|V(t)\|_F^2 + \frac{\rho}{2}\|D(t)-A(t) \\
&\qquad + W(t)\|_F^2 - \frac{\rho}{2}\|W(t)\|_F^2.
\end{aligned}
$$

The iterations are as follows:

$$
\begin{cases}
A(t)^{k+1} = [(Y(t+1)-Y(t))\cdot Y(t)^T + \rho^k(B(t)^k+U(t)^k+C(t)^k+V(t)^k+D(t)^k+W(t)^k)]\cdot[Y(t)Y(t)^T+3\rho^k I]^{-1} \\
B(t)^{k+1} = S_{\alpha/\rho^k}(A(t)^k - U(t)^k) \\
U(t)^{k+1} = U(t)^k + B(t)^k - A(t)^k \\
C(t)^{k+1} = S_{\beta/\rho^k}(A(t)^k - V(t)^k - A(t-1)^k) + A(t-1)^k \\
V(t)^{k+1} = V(t)^k + C(t)^k - A(t)^k \\
D(t)^{k+1} = S_{\beta/\rho^k}(A(t)^k - W(t)^k - A(t+1)^k) + A(t+1)^k \\
W(t)^{k+1} = W(t)^k + D(t)^k - A(t)^k
\end{cases}.
$$

Subproblem 3 is similar to subproblem 1. When $t = N-1$,

$$
\begin{cases}
A(N-1)^{k+1} = [(Y(N)-Y(N-1))\cdot Y(N-1)^T + \rho^k(B(N-1)^k+U(N-1)^k+C(N-1)^k+V(N-1)^k)]\cdot[Y(N-1)Y(N-1)^T+2\rho^k I]^{-1} \\
B(N-1)^{k+1} = S_{\alpha/\rho^k}(A(N-1)^k - U(N-1)^k) \\
U(N-1)^{k+1} = U(N-1)^k + B(N-1)^k - A(N-1)^k \\
C(N-1)^{k+1} = S_{\beta/\rho^k}(A(N-1)^k - V(N-1)^k - A(N-2)^k) + A(N-2)^k \\
V(N-1)^{k+1} = V(N-1)^k + C(N-1)^k - A(N-1)^k
\end{cases}.
$$

With some adjustments to the ADMM described above, one can use the PADMM to achieve a faster computation speed. Proper preconditioning processes are applied for the computation of $A(t), 1 \le t \le N-1$.

The form of the iterations of $A(t), 1 \le t \le N-1$, arises from $\frac{\partial L_\rho}{\partial A(t)} = 0$. Consider $t = 1$ (subproblem 1) as an example.

$$
\begin{aligned}
&\frac{\partial L_\rho(A(1),B(1),D(1),U(1),W(1))}{\partial A(1)} \\
&\qquad = A(1)\cdot[Y(1)Y(1)^T+2\rho I] - (Y(2)-Y(1))\cdot Y(1)^T - \rho(B(1)+U(1) \\
&\qquad + D(1)+W(1)) = 0
\end{aligned}
$$

is equivalent to

$$
\begin{aligned}
&A(1)\cdot[Y(1)Y(1)^T+2\rho I] \\
&\qquad = (Y(2)-Y(1))\cdot Y(1)^T + \rho(B(1)+U(1)+D(1)+W(1)).
\end{aligned}
$$

Usually, in the ADMM, the update $A(1)$ of takes the form

$$
\begin{aligned}
A(1) = [(Y(2)-Y(1))\cdot Y(1)^T + \rho(B(1)+U(1)+D(1)+W(1))]\cdot[Y(1)Y(1)^T \\
+ 2\rho I]^{-1}.
\end{aligned}
$$

With the proposed preconditioning, ,we add $-2\rho A(1)$ to both sides of Eq~(\ref{eq11}). As the result,

$$A(1) = [(Y(2) - Y(1)) \cdot Y(1)^T + \rho(B(1) + U(1) + D(1) + W(1) - 2A(1))] \cdot [Y(1)Y(1)^T]^+,$$

where $(M)^+$ denotes the general inverse of the matrix M, in case M is singular.

Similarly, we can obtain the PADMM iterations of $A(t)$,$1 \leq t \leq N-1$ for all subproblems as follows:

$$A(t)^{k+1} = \begin{cases} [(Y(t+1) - Y(t)) \cdot Y(t)^T + \rho(B(t)^k + U(t)^k + D(t)^k + W(t)^k - 2A(t)^k)] \cdot [Y(t)Y(t)^T]^+, t = 1 \\ [(Y(t+1) - Y(t)) \cdot Y(t)^T + \rho(B(t)^k + U(t)^k + C(t)^k + V(t)^k + D(t)^k + W(t)^k - 3A(t)^k)] \cdot [Y(t)Y(t)^T]^+, 1 < t < N \\ [(Y(t) - Y(t-1)) \cdot Y(t-1)^T + \rho(B(t-1)^k + U(t-1)^k + C(t-1)^k + V(t-1)^k - 2A(t-1)^k)] \cdot [Y(t-1)Y(t-1)^T]^+, t = N \end{cases}$$

The $[Y(t)Y(t)^T]^+, 1 \leq t \leq N,$ are unchanged in all iterations; therefore, they can be stored as constants. Hence, the PADMM can save N matrix inversion computations in every iteration except the first. Singular value decomposition is used to compute the general inverses of the $[Y(t)Y(t)^T]^+, 1 \leq t \leq N$. Proper preconditioning makes the computation of the matrix inverses easier while maintaining an equivalent precision. Details on the theoretical results can be found in [2].

[1] Boyd S CE Parikh N. Distributed Optimization and Statistical Learning via the Alternating Direction Method of Multipliers[J]. Foundations & Trends in Machine Learning. 2010;. s

[2] Jiao Y, Jin Q, Lu X, Wang W. Preconditioned alternating direction method of multipliers for inverse problems with constraints. Inverse Problems. 2017;33(2):025004.
