# Supplementary material for: scPADGRN: A preconditioned ADMM approach for reconstructing dynamic gene regulatory network using single-cell RNA sequencing data
Source: PLoS Comput Biol. 2020 Jul 27;16(7):e1007471. doi: 10.1371/journal.pcbi.1007471 (PMC7410337; doi:10.1371/journal.pcbi.1007471)
Supplement: S3 Text — Brief introduction to scPADGRN package and codes. (PDF) [file pcbi.1007471.s003.pdf]

S3 Text: R package scPADGRN

We provide R package scPADGRN at github: https://github.com/xzheng-ac/scPADGRN. Specific script and examples also available through the link above.

This repository contains R codes to implement scPADGRN and three demo datasets with their pre-processing scripts in the directory of \scPADGRN\data.
