# Supplementary figures and images for: scPADGRN: A preconditioned ADMM approach for reconstructing dynamic gene regulatory network using single-cell RNA sequencing data
Source: PLoS Comput Biol. 2020 Jul 27;16(7):e1007471. doi: 10.1371/journal.pcbi.1007471 (PMC7410337; doi:10.1371/journal.pcbi.1007471)

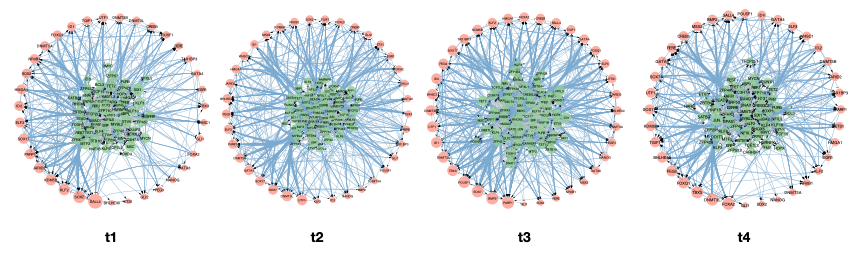

Supplement: S1 Fig — Pink nodes are differentiation-related genes and green nodes are other genes. Node size is proportional to node degree. Links among differentiation-related genes, and between differentiation-related genes and other genes are blue; links among other genes are grey. Arrow stands for activation relationship and ‘T’ stands for suppression relationship. (PNG) [file pcbi.1007471.s004.png]

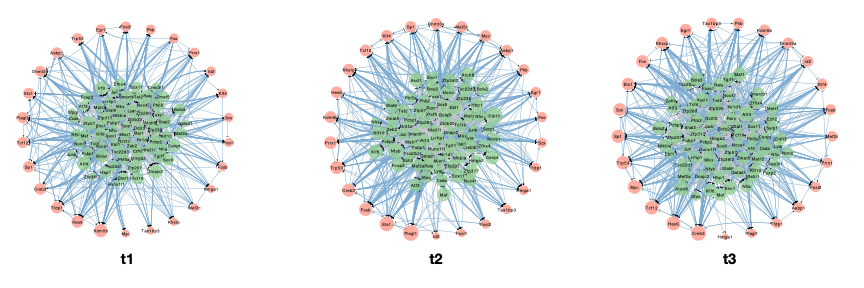

Supplement: S2 Fig — Pink nodes are differentiation-related genes and green nodes are other genes. Node size is proportional to node degree. Links among differentiation-related genes, and between differentiation-related genes and other genes are blue; links among other genes are grey. Arrow stands for activation relationship and ‘T’ stands for suppression relationship. (PNG) [file pcbi.1007471.s005.png]

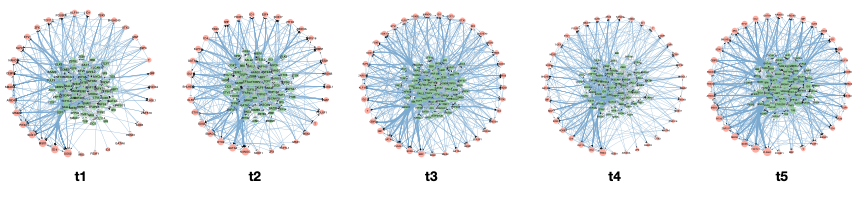

Supplement: S3 Fig — Pink nodes are differentiation-related genes and green nodes are other genes. Node size is proportional to node degree. Links among differentiation-related genes, and between differentiation-related genes and other genes are blue; links among other genes are grey. Arrow stands for activation relationship and ‘T’ stands for suppression relationship. (PNG) [file pcbi.1007471.s006.png]

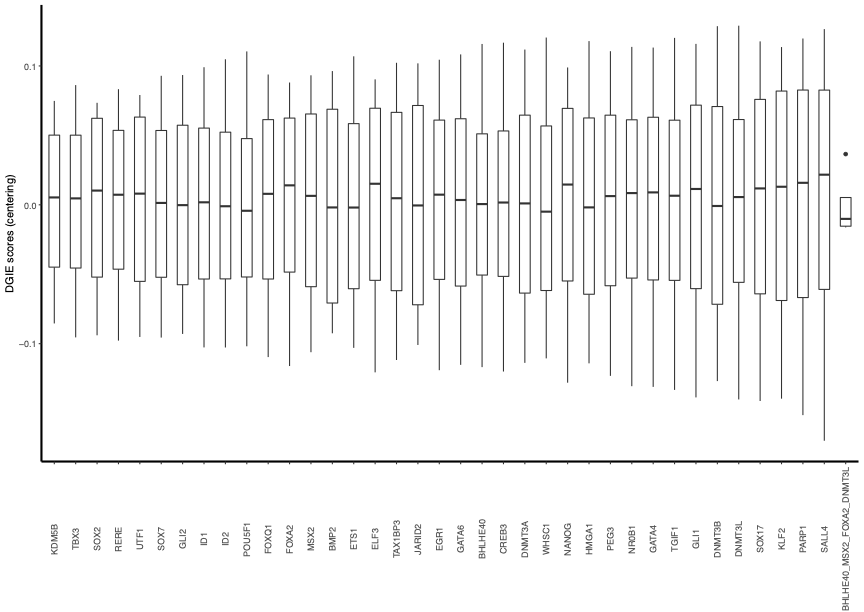

Supplement: S4 Fig — Four genes, BHLHE40, MSX2, FOXA2 and DNMT3L are identified as key regulators. (PNG) [file pcbi.1007471.s007.png]

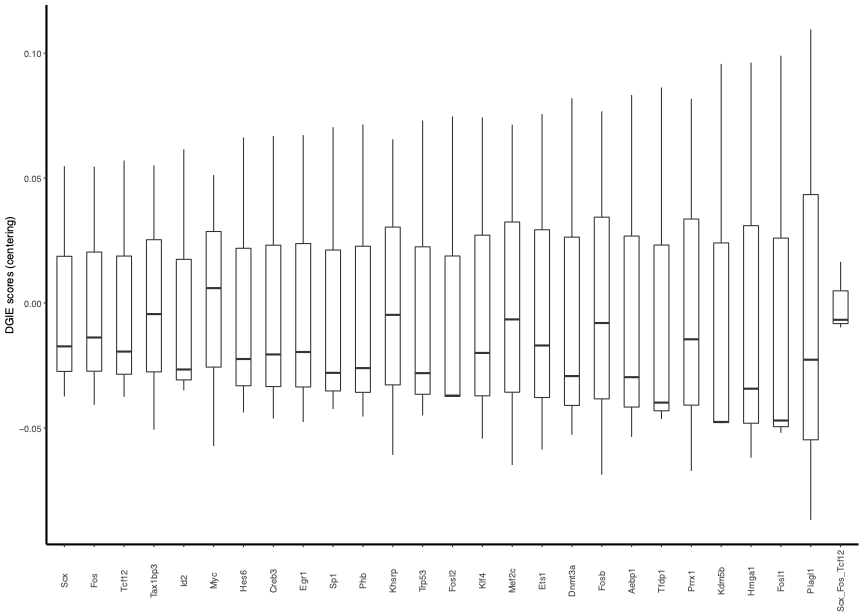

Supplement: S5 Fig — Three genes, Scx, Fos and Tcf12 are identified as key regulators. (PNG) [file pcbi.1007471.s008.png]

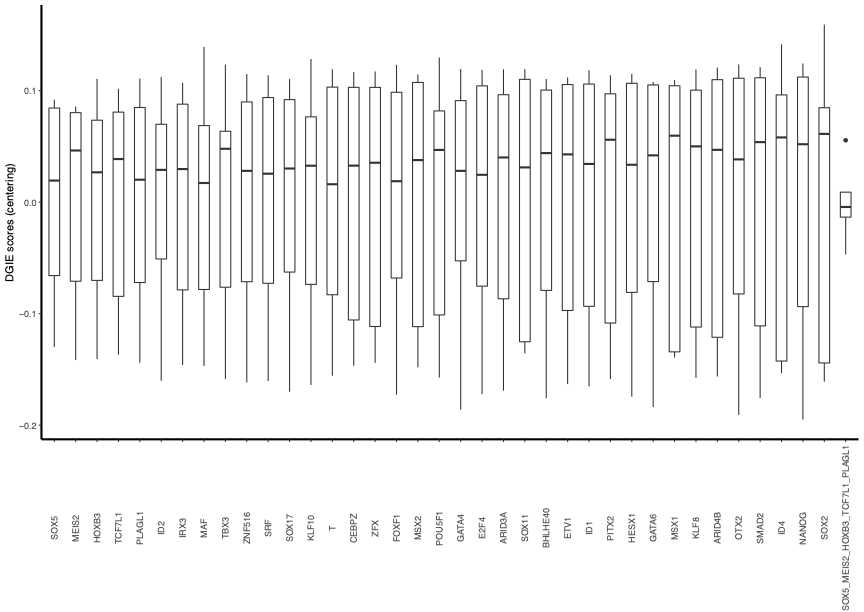

Supplement: S6 Fig — Five genes, Sox5, Meis2, Hoxb3, Tcf7l1 and Plagl1 are identified as key regulators. (PNG) [file pcbi.1007471.s009.png]

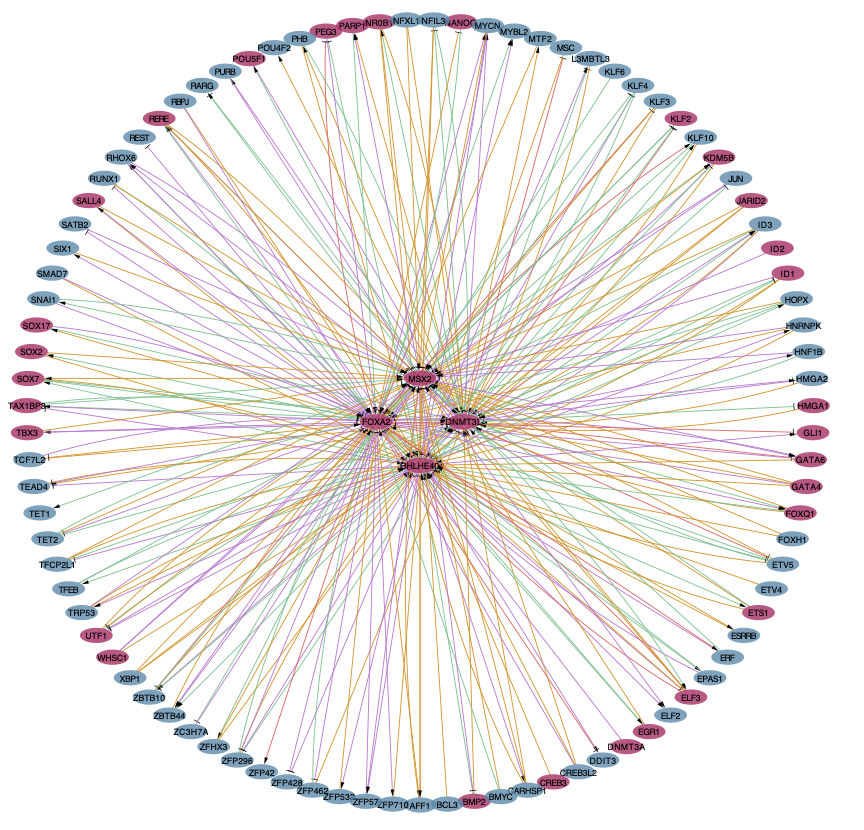

Supplement: S7 Fig — Differential network of identified targets for dataset 1. Purple nodes stand for differentiation related genes and blue nodes stand for other genes. Red links are interactions which appear at t1 exclusively. Yellow links are interactions that only exist at t2. Green links are interactions that only exist at t3. Purple links are interactions that only exist at t4. (PNG) [file pcbi.1007471.s010.png]

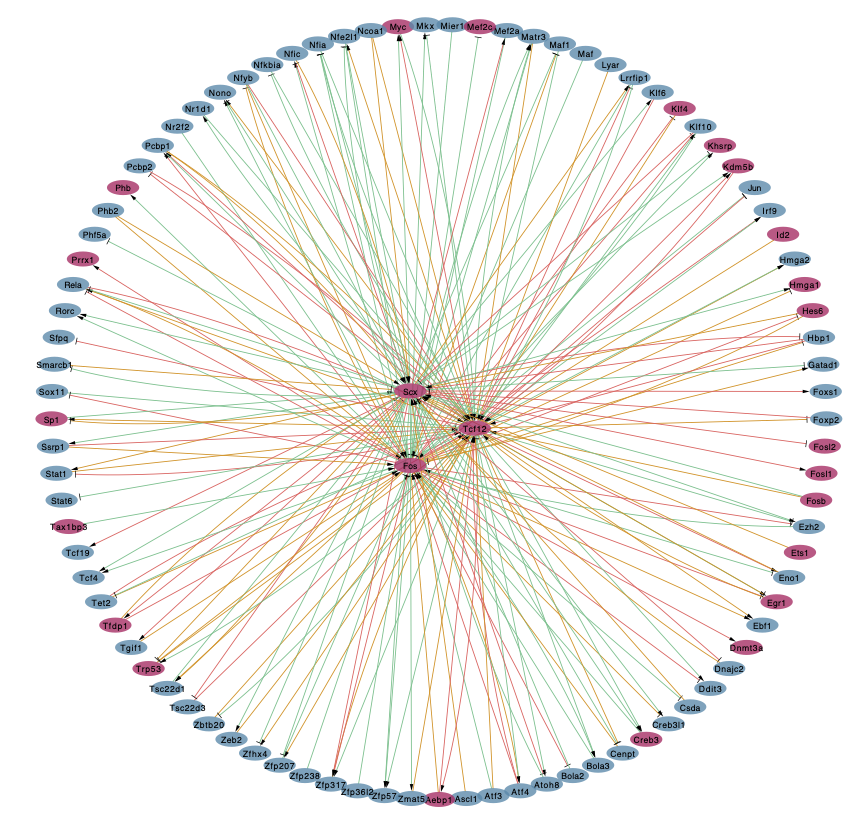

Supplement: S8 Fig — Purple nodes stand for differentiation-related genes and blue nodes stand for other genes. Red links are interactions which appear at t1 exclusively. Yellow links are interactions that only exist at t2. Green links are interactions that only exist at t3. (PNG) [file pcbi.1007471.s011.png]

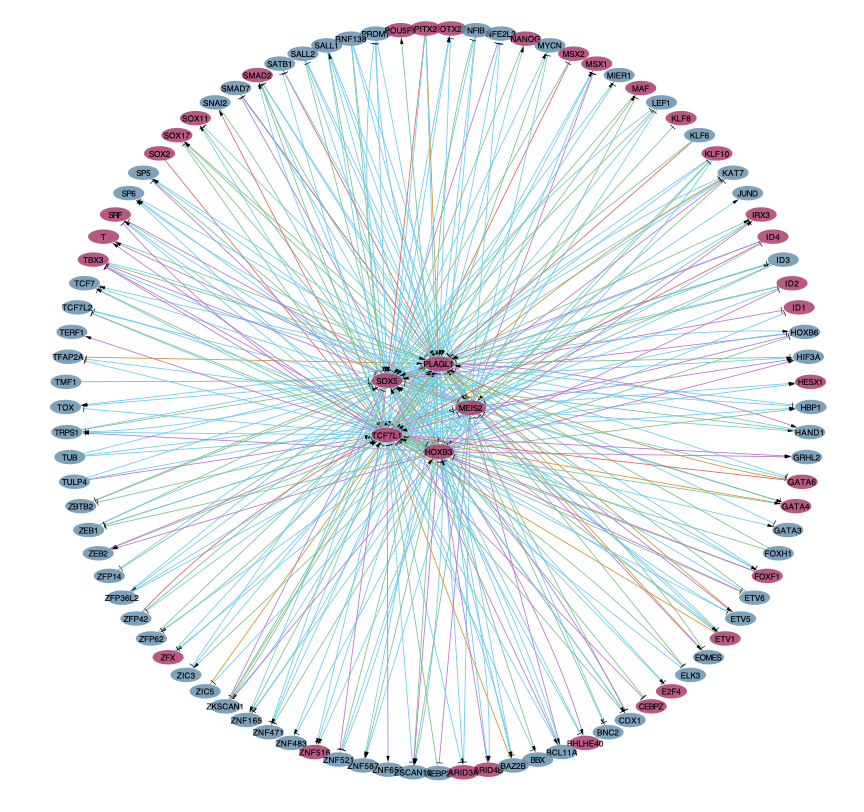

Supplement: S9 Fig — Purple nodes stand for differentiation-related genes and blue nodes stand for other genes. Red links are interactions which appear at t1 exclusively. Yellow links are interactions that only exist at t2. Green links are interactions that only exist at t3. Purple links are interactions that only exist at t4. Blue links are interactions that only exist at t5. (PNG) [file pcbi.1007471.s012.png]
