# Supplementary material for: scPADGRN: A preconditioned ADMM approach for reconstructing dynamic gene regulatory network using single-cell RNA sequencing data
Source: PLoS Comput Biol. 2020 Jul 27;16(7):e1007471. doi: 10.1371/journal.pcbi.1007471 (PMC7410337; doi:10.1371/journal.pcbi.1007471)
Supplement: S1 Table — In the first simulation experiment, we set the number of genes to 100, 200, 300, 400 and 500, individually. (PDF) [file pcbi.1007471.s013.pdf]

S1 Table: Full simulation results for Table 1

gene=100, cluster=gene, cell=10*gene, relative error=0.01													
Noise variance	Method	Time(s)	PADMM cut time by	#iteration	Reconstruction error				AUC			
					t1	t2	t3	t4	t1	t2	t3	t4
0.000	ADMM	1.244	59.60%	24	0.996	1.070	1.274	1.906	1.000	1.000	1.000	1.000
	PADMM	0.503		31	0.996	1.069	1.274	1.906	1.000	1.000	1.000	1.000
0.010	ADMM	1.167	60.81%	25	2.476	4.511	4.712	4.502	1.000	1.000	1.000	1.000
	PADMM	0.457		31	2.479	4.540	4.753	4.510	1.000	1.000	1.000	1.000
0.020	ADMM	1.216	64.00%	25	4.476	7.732	11.701	12.659	1.000	1.000	1.000	1.000
	PADMM	0.438		29	4.482	7.766	11.787	12.734	1.000	1.000	1.000	1.000
0.050	ADMM	1.180	65.55%	25	10.502	19.203	26.031	24.795	0.999	0.998	0.993	0.992
	PADMM	0.407		28	10.515	19.269	26.215	24.872	0.999	0.998	0.993	0.992

gene=200, cluster=gene, cell=10*gene, relative error=0.01													
Noise variance	Method	Time(s)	PADMM cut time by	#iteration	Reconstruction error				AUC			
					t1	t2	t3	t4	t1	t2	t3	t4
0.000	ADMM	9.056	60.64%	25	1.533	1.828	2.996	4.172	1.000	1.000	1.000	1.000
	PADMM	3.565		33	1.533	1.828	2.996	4.171	1.000	1.000	1.000	1.000
0.010	ADMM	8.746	64.48%	25	3.663	11.127	13.963	14.550	1.000	1.000	1.000	1.000
	PADMM	3.107		30	3.664	11.207	14.070	14.619	1.000	1.000	1.000	1.000
0.020	ADMM	8.013	61.27%	25	6.727	18.036	23.042	26.043	1.000	1.000	1.000	1.000
	PADMM	3.104		30	6.730	18.123	23.190	26.154	1.000	1.000	1.000	1.000
0.050	ADMM	8.036	64.99%	25	32.052	36.021	50.350	59.935	0.999	0.996	0.986	0.978
	PADMM	2.813		27	32.165	36.136	50.595	60.165	0.999	0.996	0.986	0.978

gene=300, cluster=gene, cell=10*gene, relative error=0.01													
Noise variance	Method	Time(s)	PADMM cut time by	#iteration	Reconstruction error				AUC			
					t1	t2	t3	t4	t1	t2	t3	t4
0.000	ADMM	24.676	60.07%	24	2.000	3.420	4.815	6.270	0.998	1.000	1.000	1.000
	PADMM	9.854		32	2.000	3.420	4.815	6.270	0.998	1.000	1.000	1.000
0.010	ADMM	23.896	62.31%	25	8.595	16.626	18.136	23.125	0.998	1.000	1.000	1.000
	PADMM	9.006		30	8.608	16.744	18.235	23.220	0.998	1.000	1.000	1.000
0.020	ADMM	23.952	64.47%	25	16.402	37.052	41.270	38.369	0.998	1.000	1.000	1.000
	PADMM	8.511		28	16.422	37.328	41.522	38.461	0.998	1.000	1.000	1.000
0.050	ADMM	23.925	64.26%	25	53.181	81.860	69.184	71.881	0.994	0.985	0.992	0.990
	PADMM	8.550		28	53.430	82.395	69.383	72.058	0.994	0.984	0.992	0.990

gene=400, cluster=gene, cell=10*gene, relative error=0.01													
Noise variance	Method	Time(s)	PADMM cut time by	#iteration	Reconstruction error				AUC			
					t1	t2	t3	t4	t1	t2	t3	t4
0.000	ADMM	82.376	74.23%	37	3.296	4.398	6.349	8.207	0.998	1.000	1.000	1.000
	PADMM	21.231		32	3.296	4.398	6.349	8.207	0.998	1.000	1.000	1.000
0.010	ADMM	80.076	75.06%	37	11.558	18.081	30.625	29.255	0.998	1.000	1.000	1.000
	PADMM	19.971		31	11.557	18.079	30.617	29.254	0.998	1.000	1.000	1.000
0.020	ADMM	56.072	66.64%	26	35.128	45.856	50.624	57.776	0.996	1.000	1.000	1.000
	PADMM	18.704		28	35.222	45.990	50.763	57.839	0.996	1.000	1.000	1.000
0.050	ADMM	54.215	66.46%	25	65.441	90.409	111.102	116.696	0.995	0.994	0.984	0.979
	PADMM	18.184		28	65.618	90.918	111.665	117.085	0.995	0.994	0.983	0.979

gene=500, cluster=gene, cell=10*gene, relative error=0.01													
Noise variance	Method	Time(s)	PADMM cut time by	#iteration	Reconstruction error				AUC			
					t1	t2	t3	t4	t1	t2	t3	t4
0.000	ADMM	134.612	74.12%	34	3.486	4.881	7.242	9.316	0.999	1.000	1.000	1.000
	PADMM	34.831		32	3.486	4.881	7.242	9.316	0.999	1.000	1.000	1.000
0.010	ADMM	156.080	76.18%	34	9.877	25.193	33.505	41.704	0.999	1.000	1.000	1.000
	PADMM	37.173		30	9.877	25.187	33.494	41.693	0.999	1.000	1.000	1.000
0.020	ADMM	138.125	74.29%	34	37.224	45.211	68.268	83.807	0.998	1.000	1.000	0.999
	PADMM	35.510		30	37.212	45.196	68.250	83.788	0.998	1.000	1.000	0.999
0.050	ADMM	105.719	66.46%	26	64.804	70.041	130.689	158.855	0.995	1.000	0.988	0.972
	PADMM	35.457		28	64.871	70.079	130.933	158.895	0.995	1.000	0.988	0.972
