# Supplementary material for: scPADGRN: A preconditioned ADMM approach for reconstructing dynamic gene regulatory network using single-cell RNA sequencing data
Source: PLoS Comput Biol. 2020 Jul 27;16(7):e1007471. doi: 10.1371/journal.pcbi.1007471 (PMC7410337; doi:10.1371/journal.pcbi.1007471)
Supplement: S2 Table — In the second simulation experiment, we set the number of genes to 200 and 400. (PDF) [file pcbi.1007471.s014.pdf]

S2 Table: Full simulation results for Table 2

gene=200, cluster=40,80,120,160,200, cell=10*gene, relative error=0.01													
Gene number	Cluster number	Method	Time(s)	PADMM cut time by	#iteration	Reconstruction error				AUC			
						t1	t2	t3	t4	t1	t2	t3	t4
200	200	ADMM	8.921	56.44%	25	1.533	1.828	2.996	4.172	1.000	1.000	1.000	1.000
		PADMM	3.886		33	1.533	1.828	2.996	4.171	1.000	1.000	1.000	1.000
	160	ADMM	5.819	61.57%	17	8.990	8.627	8.852	9.023	1.000	1.000	1.000	1.000
		PADMM	2.236		18	8.990	8.627	8.852	9.023	1.000	1.000	1.000	1.000
	120	ADMM	5.080	74.97%	17	12.748	12.541	12.573	12.523	1.000	1.000	1.000	1.000
		PADMM	1.272		12	12.749	12.542	12.573	12.523	1.000	1.000	1.000	1.000
	80	ADMM	5.317	72.24%	17	15.460	15.614	15.329	15.408	0.995	0.998	0.997	0.999
		PADMM	1.476		10	15.460	15.614	15.329	15.408	0.995	0.998	0.997	0.999
	40	ADMM	4.982	69.71%	17	17.658	17.777	17.812	17.784	0.970	0.979	0.984	0.984
		PADMM	1.509		10	17.658	17.777	17.812	17.784	0.970	0.979	0.984	0.984
gene=400, cluster=40*(1:10) cell=10*gene, relative error=0.01													
Gene number	Cluster number	Method	Time(s)	PADMM cut time by	#iteration	Reconstruction error				AUC			
						t1	t2	t3	t4	t1	t2	t3	t4
400	400	ADMM	80.597	74.10%	37	3.296	4.398	6.349	8.207	0.998	1.000	1.000	1.000
		PADMM	20.873		32	3.296	4.398	6.349	8.207	0.998	1.000	1.000	1.000
	360	ADMM	39.954	60.40%	19	12.541	12.559	12.576	12.592	0.998	1.000	1.000	1.000
		PADMM	15.820		24	12.541	12.559	12.575	12.591	0.998	1.000	1.000	1.000
	320	ADMM	33.953	72.98%	17	17.902	17.760	17.815	17.486	0.996	1.000	1.000	1.000
		PADMM	9.173		14	17.903	17.760	17.816	17.486	0.996	1.000	1.000	1.000
	280	ADMM	32.622	77.49%	17	21.863	21.709	21.786	22.056	0.995	1.000	1.000	1.000
		PADMM	7.342		11	21.864	21.710	21.787	22.056	0.995	1.000	1.000	1.000
	240	ADMM	32.649	80.02%	17	25.405	25.317	25.246	25.231	0.992	1.000	1.000	1.000
		PADMM	6.525		9	25.406	25.319	25.246	25.232	0.992	1.000	1.000	1.000
	200	ADMM	30.353	80.68%	17	28.151	28.170	28.234	28.193	0.993	1.000	1.000	1.000
		PADMM	5.865		8	28.153	28.171	28.234	28.193	0.993	1.000	1.000	1.000
	160	ADMM	29.650	82.12%	17	30.804	30.853	30.709	30.901	0.991	0.999	0.999	0.999
		PADMM	5.303		8	30.805	30.853	30.709	30.901	0.991	0.999	0.999	0.999
	120	ADMM	28.932	83.54%	17	33.323	33.241	33.268	33.307	0.987	0.997	0.997	0.997
		PADMM	4.762		7	33.325	33.241	33.268	33.307	0.987	0.997	0.997	0.997
	80	ADMM	28.018	83.71%	17	35.725	35.632	35.626	35.577	0.973	0.991	0.989	0.991
		PADMM	4.565		7	35.726	35.633	35.626	35.577	0.973	0.991	0.989	0.991
	40	ADMM	27.477	84.02%	17	37.809	37.791	37.725	37.894	0.926	0.957	0.964	0.949
		PADMM	4.392		7	37.810	37.791	37.725	37.894	0.926	0.957	0.964	0.949
