# Supplementary material for: scPADGRN: A preconditioned ADMM approach for reconstructing dynamic gene regulatory network using single-cell RNA sequencing data
Source: PLoS Comput Biol. 2020 Jul 27;16(7):e1007471. doi: 10.1371/journal.pcbi.1007471 (PMC7410337; doi:10.1371/journal.pcbi.1007471)
Supplement: S3 Table — Each dataset includes 100 genes. (PDF) [file pcbi.1007471.s015.pdf]

S3 Table: Gene lists in dataset 1-3

Dataset1							
SOX2	ZFP710	PEG3	RHOX6	TCF15	GLI1	CARHSP1	TCF7L2
ESRRB	MYCN	ID2	ID3	ELF3	FOXD3	TGIF1	ZFP532
UTF1	BCL3	RBPJ	ZFP428	NANOG	ETV4	SIX1	ZBTB44
EPAS1	GATA6	KDM5B	REST	HMGA1	MYBL2	RARG	ELF2
FOXQ1	HOPX	EGR1	PURB	ETV5	TEAD4	ZBTB10	ETS1
KLF2	ID1	PARP1	PHB	CREB3L2	NFIL3	XBP1	JUN
GATA4	HNF1B	BHLHE40	ZC3H7A	FOXH1	HMGA2	ZFHX3	BMYC
SOX17	L3MBTL3	RUNX1	ERF	NFXL1	GLI2	DNMT3B	POU4F2
NR0B1	KLF3	FOXA2	TET2	SOX7	MSX2	RERE	TFEB
ZFP42	DNMT3L	SNAI1	SATB2	TAX1BP3	WHSC1	MSC	DDIT3
POU5F1	SMAD7	KLF6	ZFP296	TET1	KLF4	TRP53	SALL4
DNMT3A	KLF10	BMP2	AFF1	JARID2	MTF2	ZFP57	ZFP462
TFCP2L1	TBX3	CREB3	HNRNPK				
Dataset2							
HMGA2	PHB2	SMARCB1	FOSL2	NCOA1	MEF2A	TET2	PCBP2
ASCL1	SCX	PHB	GATAD1	ETS1	CREB3	EZH2	MIER1
FOS	BOLA3	JUN	DNAJC2	ZFP238	NONO	DDIT3	KLF4
ATF3	ATF4	AEBP1	FOXS1	PRRX1	NOC4L	HBP1	RELA
HMGA1	ZBTB20	PHF5A	MEF2C	LRRFIP1	TAX1BP3	MYC	MKX
ENO1	CSDA	PLAGL1	KDM5B	KLF6	CENPT	TCF19	JUNB
HES6	TCF12	NFKBIA	NFE2L1	EBF1	MAF1	NFIC	NR2F2
TRP53	FOXP2	SNAI2	PCBP1	FOSL1	SP1	SSRP1	DNMT3A
EGR1	TCF4	TSC22D3	IRF9	TFDP1	STAT1	ZMAT5	RUNX1T1
ZFP36L2	MATR3	TGIF1	KHSRP	ID2	SFPQ	SOX9	KLF10
SOX11	FUBP1	FOSB	NFYB	ZFP207	ZFP317	ZFP260	ZEB2
ZFP57	TSC22D1	RORC	NFIA	BOLA2	NR1D1	STAT6	ZFHX4
LYAR	ATOH8	MAF	CREB3L1				
Dataset3							
GATA6	SOX5	NFE2L3	ZNF471	NFIB	KLF10	AEBP2	SMAD7
NANOG	ZFP36L2	ZEB1	ARID4B	SOX2	ZIC5	CDX1	ZNF587
T	LEF1	FOXF1	SNAI2	MSX1	BBX	TCF7L1	SOX17
EOMES	BCL11A	PITX2	ZNF483	KLF8	RNF138	MIER1	ZFP62
ID2	HBP1	HOXB3	MAF	GRHL2	SATB1	KLF6	SRF
PRDM1	IRX3	ID4	ETV6	ZNF521	BAZ2B	MYCN	JUND
ID1	MEIS2	ZNF165	ZKSCAN1	BHLHE40	ELK3	SMAD2	TUB
ZNF516	ZFX	TCF7L2	ETV1	TFAP2A	ZBTB2	PLAGL1	ZFP14
SHOX2	TERF1	POU5F1	TMF1	GATA3	ETV5	ZSCAN10	ARID3A

TBX3	TOX	SP5	HIF3A	ZEB2	SP6	ZNF652	CEBPZ
GATA4	FOXH1	ZIC3	SALL2	BNC2	ZFP42	HOXB6	E2F4
OTX2	HESX1	TRPS1	SHOX	KAT7	TULP4	MSX2	TCF7
HAND1	SOX11	ID3	SALL1				
