# Supplementary material for: scPADGRN: A preconditioned ADMM approach for reconstructing dynamic gene regulatory network using single-cell RNA sequencing data
Source: PLoS Comput Biol. 2020 Jul 27;16(7):e1007471. doi: 10.1371/journal.pcbi.1007471 (PMC7410337; doi:10.1371/journal.pcbi.1007471)
Supplement: S4 Table — Tt(k) is the set of genes with the top k largest degree in the DGRN at time t. V1 is the set of differentiation-related genes. |V(1)||V| is the reference rate defined by the ratio of differentiation-related genes to all genes. |V(1)∩Tt(k)||Tt(k)| is the rate of differentiation-related genes among genes with the top k largest degree nodes. (PDF) [file pcbi.1007471.s016.pdf]

S4 Table: Rate comparison between rate $\frac{|V_{(1)} \cap T_t^{(k)}|}{|T_t^{(k)}|}$, $k = 10{,}50$ and reference rate $\frac{|V_{(1)}|}{|V|}$. $T_t^{(k)}$ is the set of genes with the top k largest degree in the DGRN at time t. V_1 is the set of differentiation-related genes. $\frac{|V_{(1)}|}{|V|}$ is the reference rate defined by the ratio of differentiation-related genes to all genes. $\frac{|V_{(1)} \cap T_t^{(k)}|}{|T_t^{(k)}|}$ is the rate of differentiation-related genes among genes with the top k largest degree nodes.

S4 Table (A): Rate comparison for dataset 1

$\frac{\lvert V_{(1)}\rvert}{\lvert V\rvert}$	k	$\frac{\lvert V_{(1)} \cap T_t^{(k)}\rvert}{\lvert T_t^{(k)}\rvert}$			
		t1	t2	t3	t4
0.37	10	0.4	0.6	0.7	0.6
	50	0.32	0.46	0.42	0.52

S4 Table (B): Rate comparison for dataset 2

$\frac{\lvert V_{(1)}\rvert}{\lvert V\rvert}$	k	$\frac{\lvert V_{(1)} \cap T_t^{(k)}\rvert}{\lvert T_t^{(k)}\rvert}$		
		t1	t2	t3
0.26	10	0.1	0.2	0.3
	50	0.24	0.16	0.24

S4 Table (C): Rate comparison for dataset 3

$\frac{\lvert V_{(1)}\rvert}{\lvert V\rvert}$	k	$\frac{\lvert V_{(1)} \cap T_t^{(k)}\rvert}{\lvert T_t^{(k)}\rvert}$				
		t1	t2	t3	t4	t5
0.37	10	0.5	0.3	0.2	0.4	0.4
	50	0.34	0.3	0.4	0.4	0.44
