# Supplementary material for: scPADGRN: A preconditioned ADMM approach for reconstructing dynamic gene regulatory network using single-cell RNA sequencing data
Source: PLoS Comput Biol. 2020 Jul 27;16(7):e1007471. doi: 10.1371/journal.pcbi.1007471 (PMC7410337; doi:10.1371/journal.pcbi.1007471)
Supplement: S5 Table — In the estimated differential networks, this table shows counts of links. (PDF) [file pcbi.1007471.s017.pdf]

Table S5: Number of links and confirmed links in the estimated differential networks.

Dataset	Number of links	Number of confirmed links
Dataset 1	283	28
Dataset 2	209	24
Dataset 3	318	9
